# How to measure distance visual acuity

**Published:** 2014

**Authors:** Janet Marsden, Sue Stevens, Anne Ebri

**Affiliations:** Nurse Advisor: *Community Eye Health Journal*, London, UK. Email: J.Marsden@mmu.ac.uk; Nurse Advisor (retired): *Community Eye Health Journal.*; West Africa Sub-Regional Manager: Brien Holden Vision Institute, Calabar, Nigeria.

**Figure:**
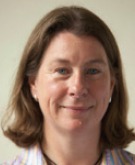
Janet Marsden

**Figure:**
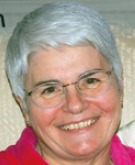
Sue Stevens

**Figure:**
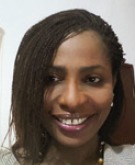
Anne Ebri

Visual acuity (VA) is a measure of the ability of the eye to distinguish shapes and the details of objects at a given distance. It is important to assess VA in a consistent way in order to detect any changes in vision. One eye is tested at a time.

## Indications

- To provide a baseline recording of VA
- To aid examination and diagnosis of eye disease or refractive error
- To assess any changes in vision
- To measure the outcomes of cataract or other surgery.

## Equipment

- Multi-letter Snellen or E chart
- Plain occluder, card or tissue
- Pinhole occluder
- Torch or flashlight
- Patient's documentation.

## Procedure

- Ensure good natural light or illumination on the chart. It is important to ensure that the person has the best possible chance of seeing and reading the test chart as treatment decisions are made based on the results of VA testing.
- If the test is done outdoors, the chart should be in bright light and the patient in the shade, with enough light to illuminate the patient's face during the test.
- Explain the procedure to the patient. Tell patients that it is not a test that they have to pass, but a test to help us know how their eyes are working. Tell them not to guess if they cannot see.
- Ensure that any equipment that the patient touches is clean and is cleaned between patients. Infections can be passed between patients if equipment – or the testers' hands – are not clean.
- Position the patient, sitting or standing, at a distance of 6 metres from the chart. The patient can hold one end of a cord or rope of 6 metres long to ensure that the distance is maintained
- Test the eyes one at a time, at first without any spectacles (if worn).
  **Note**: Some people prefer to always test the right eye first. Others prefer to test the ‘worse’ eye first (ask the patient out of which eye they see best). This ensures that the minimum is read with the ‘worse’ eye, and more will be read with the ‘good’ eye. This means that no letters are remembered, which could make the second visual acuity appear better than it is.
  **Figure:**
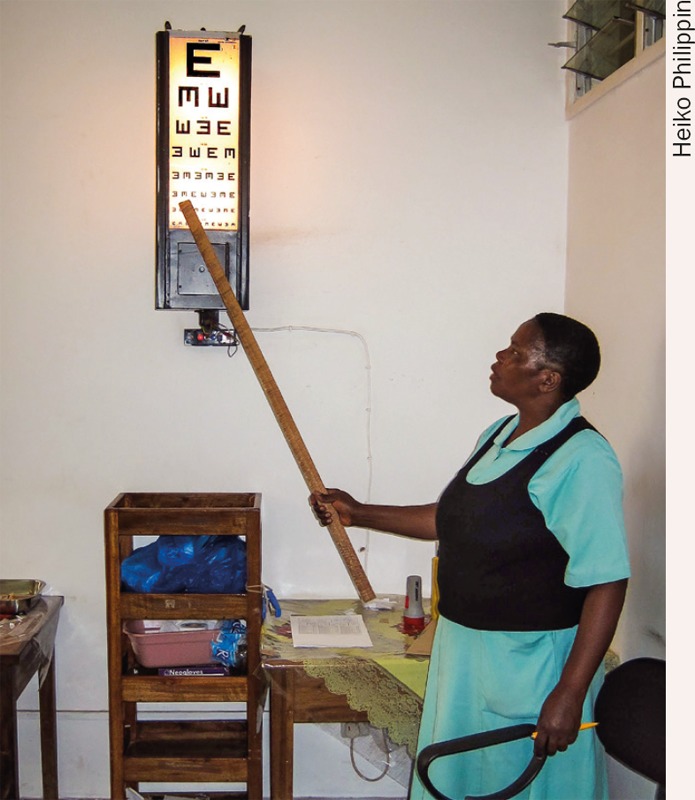
Visual acuity should be measured from a standard distance, using a standard chart with a white background
- Ask the patient to cover one eye with a plain occluder, card or tissue. They should not press on the eye; this is not good for an eye that has undergone surgery. It can also make any subsequent intraocular pressure reading inaccurate and it will distort vision when the occluded eye is tested.
- Ask the patient to read from the top of the chart and from left to right. If the patient cannot read the letters due to language difficulties, use an E chart. The patient is asked to point in the direction the ‘legs’ of the E are facing.
  **Note**: there is a one in four chance that the patient can guess the direction; therefore it is recommended that the patient should correctly indicate the orientation of most letters of the same size, e.g. four out of five or five out of six.
- The smallest line read is expressed as a fraction, e.g. 6/18. The upper number refers to the distance the chart is from the patient (6 metres) and the lower number (usually written next to the line on the chart) is the distance in metres at which a ‘normal’ eye is able to read that line of the chart.
- Incomplete lines can be added to the last complete line. e.g. 6/12+3, indicating that the patient read the ‘12’ line at 6 metres and gained three of the letters on the ‘9’ line.
- Record the VA for each eye in the patient‘s notes, stating whether it is with or without correction (spectacles). For example: **Right VA = 6/18 with correction, Left VA= 6/24 with correction.**
- If the patient cannot read the largest (top) letter at 6 metres, move him/her closer, 1 metre at a time, until the top letter can be seen – the VA will then be recorded as 5/60 or 4/60, etc.
- If the top letter cannot be read at 1 metre (1/60), hold up your fingers at varying distances of less than 1 metre and check whether the patient can count them. This is recorded as counting fingers (CF): **VA = CF**
- If the patient cannot count fingers, wave your hand and check if he/she can see this. This is recorded as hand movements (HM): **VA = HM**
- If the patient cannot see hand movements, shine a torch toward the eye and ask if they can see the light. If they can, record ‘perception of light’ (**VA = PL**). If they cannot, record ‘no perception of light’ (**VA = NPL**).
- After testing without any correction, test the patient while wearing any current distance spectacles and record the VA in each eye separately, with correction.
- If 6/6 (normal vision) is not achieved, test one eye at a time at 6 metres using a pinhole occluder (plus any current spectacles). The use of the pinhole reduces the need to focus light entering the eye.
- If the vision improves, it indicates the visual impairment is due to irregularities in the cornea, a problem in the lens, or refractive error, which is correctable with spectacles or a new prescription.
- Repeat the whole procedure for the second eye
- Summarise the VA of both eyes in the patient's notes, for example:
  **Right VA= 6/18 without specs, 6/6 with pinhole and Left VA= NPL.**

## Children

- It is usually possible, with patience, to measure VA in children from about the age of 5, although this does vary. Children need praise and reassurance that it does not matter if they are wrong.
- Ask the parent to cover the eye not being tested, so that the child cannot see around the occluder.
- Try to show only one line of the chart to the child at a time.

VA testing measures one aspect of visual function, but it is important that it is done well and accurately. An incorrect VA can lead to inappropriate decisions and management.

